# Imported Toxigenic *Corynebacterium Diphtheriae* in Refugees with Polymicrobial Skin Infections, Germany, 2022

**DOI:** 10.3201/eid2910.230285

**Published:** 2023-10

**Authors:** Benedikt Daniel Spielberger, Anna Hansel, Alea Nazary, Eva-Maria Kleißle, Claus-Georg Lehr, Marcel Utz, Juliana Hofer, Siegbert Rieg, Winfried V. Kern

**Affiliations:** University Medical Centre and Faculty of Medicine Freiburg, Freiburg, Germany

**Keywords:** Diphtheria, cutaneous diphtheria, skin infection, toxigenic *Corynebacterium diphtheria*, bacteria, refugee, refugee reception center, Germany

## Abstract

During August–December 2022, toxigenic *Corynebacterium diphtheriae* was isolated from 25 refugees with skin infections and 2 refugees with asymptomatic throat colonization at a refugee reception center in Germany. None had systemic toxin-mediated illness. Of erosive/ulcerative skin infections, 96% were polymicrobial. Erosive/ulcerative wounds in refugees should undergo testing to rule out cutaneous diphtheria.

Diphtheria is a potentially lethal upper respiratory tract infection that causes systemic illness associated with toxemia. Cases are mostly caused by toxigenic *Corynebacterium diphtheriae* and, rarely, by *C. ulcerans* through animal-to-human transmission (*1*). Although <500 cases have been detected in Europe during 2010–2019 (*2*), outbreaks have been reported in resource-limited settings (e.g., in refugee camps or in settings of waning immunization coverage) (*3*,*4*). During June–October 2022, a total of 371 diphtheria cases were detected in Europe; most (147 cases) were in Germany. Ongoing cases in 2023 and a fatal case in Belgium in June 2023 reported by the European Centre for Disease Prevention and Control (ECDC) highlight the need for further awareness (https://www.ecdc.europa.eu/en/publications-data/communicable-disease-threats-report-2-8-july-2023-week-27; https://www.ecdc.europa.eu/en/publications-data/communicable-disease-threats-report-30-january-5-february-2023-week-5; https://www.ecdc.europa.eu/en/publications-data/communicable-disease-threats-report-11-17-june-2023-week-24).

Since 2015, the University Medical Centre Freiburg has run an outpatient clinic at the refugee reception center in Freiburg, which in late 2022 detected an unusually high number of skin infections in refugees. After the initial case of cutaneous *C. diphtheriae* infection was detected, the University Medical Centre consulted with the local health authorities, and subsequent patients with skin wounds or erosive/ulcerative lesions were tested for throat colonization and skin infection with *C. diphtheriae*. Contacts of patients with confirmed cases (roommates, other close contacts) were identified and screened for *C. diphtheriae* skin infection and throat colonization. Our retrospective analysis was approved by the ethics committee of the University Medical Centre Freiburg (22-1493-S1-retro). Anonymized photographs were taken with the verbal consent of the patients. 

## The Study

In the beginning of the analysis period, we detected *C. diphtheriae* by using Columbia blood agar with fosfomycin plates and, after sufficient production, with tellurite agar. We confirmed isolates as *C. diphtheriae* by using matrix-assisted laser desorption/ionization time-of-flight mass spectrometry and using Bruker MALDI Biotyper (*5*). We detected the diphtheria toxin gene by using a conventional PCR assay (*6*). We sent all isolates for confirmation to the consiliary laboratory at Landesamt für Gesundheit Bayern (LGL Bayern, Oberschleißheim, Germany), where Elek test and multilocus-sequence typing (MLST) were additionally performed for several isolates.

During August 1, 2022, through December 31, 2022, *C. diphtheriae* was detected in 27 refugees. Of those, 25 had sought care because of nonhealing skin lesions or wounds. Screening of 154 contacts (throat swabs) identified 2 asymptomatic carriers of *C. diphtheriae* without skin lesions. All patients were male, and most were young (mean 24 years, range 18–49 years). Three patients stated that they were minors but were classified as adults by local health authorities. Most patients were born in Afghanistan (15 persons) or Syria (11 persons); 1 refugee reported Morocco as his country of birth. Of the 18 (67%) refugees who were asked about their escape route, all stated that they had fled via Balkan states (Table 1; Appendix Table 1).

**Table 1:** Demographics and clinical manifestations of 27 refugees with imported toxigenic *Corynebacterium diphtheriae* in polymicrobial skin infections, Germany, 2022*

Study population	No. (%)
Mean age at first assessment (range), y	24 (18–49)
Suspected unaccompanied minor refugee	3 (11)
Country of birth	
Afghanistan	15 (56)
Syria	11 (41)
Morocco	1 (4)
Route of escape	
Asked about route	18 (67)
Answered Balkan	18 (100)
Clinical manifstations	
Any skin ulcer	25 (93)
Lower thigh	17 (63)
Knee	3 (11)
Upper thigh	2 (7)
Feet	11 (41)
Hands	8 (30)
Genitals	2 (7)
Patients without skin wounds	2 (7)
And with *C. diphtheriae* in throat swab specimen	2 (100)
Mean duration of wounds (range), d	22.5 (3–60)

*Values are no. (%) except as indicated. All patients were male.

Cutaneous lesions persisted for a mean of 22.5 days; ≈70% persisted >14 days, and the overall range was wide, 3–60 days. Available detailed histories of the cause and circumstances of the primary manifestation indicated waterway crossings and forest habitation as the probable infection mode. The lesions were predominantly localized at the extremities: lower thigh (17 [63%]), feet and ankles (11 [41%]), hands (8 [32%]), and upper thigh (2 [7%]). In addition, genital lesions were observed in 2 refugees (7%) (Table 1; Appendix Table 1). Skin wounds appeared as partly punched-out, partly erosive lesions with erythematous margins (Figure panels A, B). Grayish mucous membranes or purulent lesions were detectable in isolated cases only. 

**Figure:**
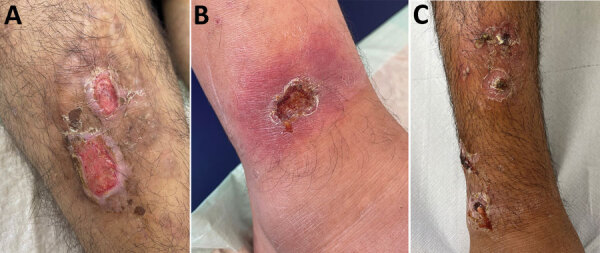
Clinical manifestations of *Corynebacterium diphtheriae* skin infections in patients at a refugee reception center in Freiburg. Germany, 2022. A) Chronic erosive skin lesions at the ventral lower thigh. Toxigenic *C. diphtheriae,* methicillin-sensitive *Staphylococcus aureus* (MSSA), and *Streptococcus pyogenes* were grown from skin swab samples. B) Ulcerative lesion with erythematous halo just above the right ankle. *C. diphtheriae*, MSSA, and *S. pyogenes* were detected from skin swab samples. C) Ecthymata at the lower leg without detection of *C. diphtheriae* but with growth of MSSA and *S. pyogenes*.

Clinical differentiation between skin infections caused by *Staphylococcus aureus*, *Streptococcus pyogenes*, or both (Figure panel C) or wounds with evidence of *C. diphtheriae* was difficult. In addition to *C. diphtheriae*, both *S. aureus* and *S. pyogenes* were detected in 21 (84%) of the 25 refugees. In 3 (12%) refugees, *C. diphtheriae* and *S. pyogenes* were detected; in 1, only *C. diphtheriae* was detected. All 25 skin infections were colonized with toxin-producing *C. diphtheriae* as shown by positive PCR for the *tox* gene or positive Elek test through the consiliary laboratory (Table 2; Appendix Table 1). The 2 cases of *C. diphtheriae* throat colonization were nontoxigenic. Among the 21 *S. aureus* isolates, 10 (48%) were methicillin resistant. Of the 24 *S. pyogenes* isolates, 3 (13%) showed resistance to clindamycin (Table 2). According to current European Committee on Antimicrobial Susceptibility Testing (https://www.eucast.org) recommendations, all *C. diphtheriae* isolates showed in vitro susceptibility to penicillin at increased exposure, and all but 2 isolates showed sensitivity to erythromycin or clindamycin (*7*). MLST was available for 16 isolates; sequence types 377 or 574 were identified 6 times, and sequence type 384 was identified 4 times. We established no correlation between country of origin and sequence type. 

**Table 2:** Microbiology test results for 27 refugees with imported toxigenic *Corynebacterium diphtheriae* in polymicrobial skin infections, Germany, 2022*

Microbiology result	No. (%)
No. *C. diphtheriae* isolates	27
Any skin wound	25 (93)
Skin wounds with toxigenic *C. diphtheriae*	25
Toxigenic *C. diphtheriae* in skin wound and throat swab	5 (20)
Any throat swab with *C. diphtheriae*	7 (26)
And without skin wounds	2 (29)
Patients without skin wounds	2 (7)
And wth *C. diphtheriae* in throat swab	2 (100)
Contact persons screened	154
Co-colonization of skin infections (% of all skin wounds)	
*C. diphtheriae***, ***Staphylococcus. aureus, Streptococcus pyogenes*	21 (84)
*C. diphtheriae***, ***S. pyogenes*, no *S. aureus*	3 (12)
*C. diphtheriae*, no *S. pyogenes*, no *S. aureus*	1 (4)
Antimicrobial resistance	
Total *S. aureus* isolates	21
MSSA	11 (52)
Community-acquired MRSA	10 (48)
Hospital-acquired MRSA	0
Total no. *S. pyogenes* isolates	24
Resistance to clindamycin	3 (13)

*Values are no. (%) except as indicated. MRSA, methicillin-resistant *S. aureus*; MSSA, methicillin-sensitive *S. aureus.*

Detection of toxin-producing *C. diphtheriae* from throat swab samples was successful in 5 (20%) of the 25 patients with cutaneous lesions. No systemic illnesses associated with toxemia were observed in our cohort.

## Conclusions

Our report adds details about the clinical picture of cutaneous diphtheria among refugees from Afghanistan, Syria, and Morocco in Germany. In this cohort, 100% of cutaneous infections were caused by toxigenic *C. diphtheriae*, which is a higher proportion than the 27% toxigenic cutaneous infections reported in 270 cases published over the past 65 years (Appendix Table 2). This magnitude indicates a common source of infection or increased risks for transmission while fleeing (*8*,*9*). 

Nearly all skin infections in this cohort were polymicrobial, caused by *C. diphtheriae*, *S. aureus*, and *S. pyogenes*. Co-infections with *S. pyogenes* have been reported in the literature for 151 (56%) cases and with *S. aureus* for 110 (41%); methicillin resistance was noted in 19 (7%) cases (Appendix Table 2). Only 1 report indicates a rate of co-pathogens in the magnitude of that found in our cohort (*10*). Similar to a previous report from Germany, the isolates were broadly drug susceptible (*11*); susceptibility to penicillin seemed to be higher than that reported from Spain during 2014–2019 (*12*).

Another finding was that one fifth of the refugees with cutaneous diphtheria were concurrently colonized with toxigenic *C. diphtheriae* in the throat while remaining systemically asymptomatic. Such concurrent throat plus skin colonization may be highly relevant for transmission and should be identified. 

Although in their rapid risk assessment the ECDC considered the overall risk for the residing population in refugee-accepting countries to be very low, several steps are crucial for preventing spread and casualties (*13*). Toxin-mediated systemic disease can be effectively prevented by universal immunization against diphtheria toxin. Refugee infants are particularly at risk because of the reported low rates of receiving a third dose of diphtheria-tetanus-pertussis vaccine in Afghanistan (81%) and Syria (48%) (*14*). Furthermore, waning immunity against vaccine-preventable diseases, especially for pertussis and diphtheria, is reported where antibody levels drop to prevaccination levels 5–6 years after vaccination (*15*). 

Our report, in conjunction with material from ECDC, could be informative for migrants and healthcare workers with regard to identifying cutaneous diphtheria (*13*). These findings supplement recommendations for contact tracing, screening for throat colonization, and using personal protective equipment when changing dressings or taking swab specimens.

Workers at refugee reception centers should pay attention to chronic erosive/ulcerative wounds in refugees. They should conduct adequate microbiological investigations to rule out cutaneous diphtheria, even if *S. aureus* or *S. pyogenes* have already been identified, and should screen persons, including contact persons, for throat colonization. Booster vaccinations or full immunizations against diphtheria toxin and antimicrobial prophylaxis should be given in accordance with ECDC guidelines in risk settings when cases of diphtheria are suspected (*13*).

Appendix Additional information for study of imported toxigenic *Corynebacterium diphtheriae* in refugees with polymicrobial skin infections, Germany, 2022.
