# Supplementary material for: Imported Toxigenic Corynebacterium Diphtheriae in Refugees with Polymicrobial Skin Infections, Germany, 2022
Source: Emerg Infect Dis. 2023 Oct;29(10):2112–5. doi: 10.3201/eid2910.230285 (PMC10521595; doi:10.3201/eid2910.230285)
Supplement: Appendix — Additional information for study of imported toxigenic Corynebacterium diphtheriae in refugees with polymicrobial skin infections, Germany, 2022. [file 23-0285-Techapp-s1.pdf]

Article DOI: https://doi.org/10.3201/eid2910.230285

EID cannot ensure accessibility for supplementary materials supplied by authors. Readers who have difficulty accessing supplementary content should contact the authors for assistance.

Imported Toxigenic *Corynebacterium Diphtheriae* in Refugees with Polymicrobial Skin Infections, Germany, 2022

Appendix

Appendix Table 1. Data for Refugees with imported toxigenic *Corynebacterium diphtheriae* in polymicrobial skin infections, Germany, 2022*

ID	Age	Umr	Country of birth	Escape route	Skin infection	Infection site	Throat swab	Skin swab	MALDI-TOF	Elek test	*tox* PCR	Agar type	Penicillin MIC [mg/l]	Penicillin EUCAST v. 13.1	Erythromycin MIC [mg/l]	Erythromycin EUCAST v. 13.1	Clindamycin EUCAST v. 13.1	Ciprofloxacin EUCAST v. 13.1	Sequence (MLST)
1	25		Afghanistan	Balkan	yes	lower thigh	MRSA	CD, MSSA, S. pyogenes	C. diphtheriae mitis	positive	positive	coblfos	0,125	I	-	S	S	I	377
2	20		Syria	nd	yes	feet, lower thigh		CD, MRSA, S. pyogenes	C. diphtheriae gravis	positive	positive	coblfos	0,064	I	0,016	S	S	I	-
3	20		Syria	nd	yes	lower thigh		CD, S. pyogenes	C. diphtheriae mitis	positive	positive	coblfos	0,19	I	0,016	S	S	I	-
4	24		Syria	Balkan (TR, MK, BG, RS, SI, CZ)	yes	lower thigh		CD, MRSA, S. pyogenes	C. diphtheriae gravis	positive	positive	coblfos	0,064	I	< 0,016	S	S	I	-
5	25		Syria	Balkan	yes	feet, lower thigh	CD	CD, MRSA, S. pyogenes	C. diphtheriae mitis	positive	positive	coblfos	0,064	I	0,016	S	S	I	-
6	23		Syria	nd	yes	feet		CD, S. pyogenes	C. diphtheriae mitis	positive	positive	coblfos	0,094	I	0,016	S	S	I	377
7	28		Syria	Balkan	yes	feet		CD, MRSA, S. pyogenes	C. diphtheriae gravis	positive	positive	coblfos	0,064	I	< 0,016	S	S	I	384
8	18		Afghanistan	Balkan (TR, BG, RS, HU)	yes	lower thigh		CD, MSSA, S. pyogenes	C. diphtheriae mitis	positive	positive	coblfos	0,19	I	0,016	S	S	I	574
9	33		Syria	Balkan (RS)	yes	lower thigh	CD	CD, MSSA, S. pyogenes	C. diphtheriae	positive	positive	coblfos	0,125	I	0,016	S	S	I	377
10	22		Syria	Balkan	yes	chin	CD	CD, S. pyogenes	C. diphtheriae	positive	-	coblfos	0,094	I	0,016	S	S	I	384

ID	Age	Umr	Country of birth	Escape route	Skin infection	Infection site	Throat swab	Skin swab	MALDI-TOF	Elek test	*tox* PCR	Agar type	Penicillin MIC [mg/l]	Penicillin EUCAST v. 13.1	Erythromycin MIC [mg/l]	Erythromycin EUCAST v. 13.1	Clindamycin EUCAST v. 13.1	Ciprofloxacin EUCAST v. 13.1	Sequence (MLST)
11	23		Syria	Balkan	yes	lower thigh	S. dysgalactiae, S. canis	CD, MSSA, S. pyogenes	C. diphtheriae	positive	positive	coblfos	0,125	I	0,023	S	S	I	377
12	49		Syria	Balkan (TR, RS, SV, AT)	yes	feet, lower thigh		CD, MRSA, S. pyogenes	C. diphtheriae	positive	-	coblfos	0,125	I	0,016	S	S	I	384
13	20		Afghanistan	nd	yes	knee, lower thigh		CD, MSSA, S. pyogenes	C. diphtheriae	positive	-	coblfos	0,125	I	0,023	S	S	I	574
14	35		Syria	nd	yes	feet, hands	MSSA	CD, MRSA, S. pyogenes, Citrobacter koseri	C. diphtheriae	positive	-	tell	0,094	I	0,016	S	S	I	384
15	22	yes	Afghanistan	Balkan (RS)	yes	feet, lower thigh, hands		CD, MSSA, S. pyogenes	C. diphtheriae	positive	-	tell	0,19	I	6	R	R	R	377
16	18		Afghanistan	nd	yes	feet, genital, hands		CD, MRSA, S. pyogenes	C. diphtheriae	positive	-	tell	0,19	I	8	R	R	R	377
17	25		Afghanistan	Balkan (MK, RS, HU, AT, CH)	yes	lower thigh, hands		CD, MSSA, S. pyogenes	C. diphtheriae	positive	-	tell	0,125	I	0,023	S	S	I	574
18	18		Afghanistan	nd	yes	ear, hands		CD, MRSA, S. pyogenes	C. diphtheriae	positive	-	tell	0,19	I	0,016	S	S	R	-
19	18	yes	Afghanistan	Balkan	yes	feet, lower thigh		CD, S. pyogenes	C. diphtheriae	-	positive	tell	-	-	-	-	S	S	-
20	18	yes	Afghanistan	nd	yes	feet, lower thigh, hands		CD, MSSA, S. pyogenes	C. diphtheriae	positive	-	tell	0,19	I	0,047	S	S	I	574
21	18		Afghanistan	Balkan (IR, TR, BG, RS, HU, AT, CH)	no	none	CD	no skin infection	C. diphtheriae	-	negative	tell	0,38	I	0,032	S	S	I	-
22	18		Afghanistan	nd	no	none	CD	no skin infection	C. diphtheriae	negative	negative	tell	0,25	I	0,016	S	S	I	-
23	20		Afghanistan	Balkan (IR, TR, BG, RS, BA, HR, IT, CH)	yes	lower thigh, knee		CD, MSSA, S. pyogenes	C. diphtheriae	positive	-	tell	0,25	I	0,023	S	S	I	574
24	33		Afghanistan	Balkan (IR, TR,	yes	feet, genital, upper thigh		CD, MSSA, S. pyogenes	C. diphtheriae	positive	-	tell	-	I	-	S	S	-	574

ID	Age	Umr	Country of birth	Escape route	Skin infection	Infection site	Throat swab	Skin swab	MALDI-TOF	Elek test	*tox* PCR	Agar type	Penicillin MIC [mg/l]	Penicillin EUCAST v. 13.1	Erythromycin MIC [mg/l]	Erythromycin EUCAST v. 13.1	Clindamycin EUCAST v. 13.1	Ciprofloxacin EUCAST v. 13.1	Sequence (MLST)
				RS, BA, IT, CH)															
25	22		Afghanistan	Balkan (BA)	yes	lower thigh, hands		CD	C. diphtheriae	-	positive	tell	-	-	-	-	-	-	-
26	27		Morocco	Balkan (GR, AL, XK, RS, BA, HR, IT, NL)	yes	hands	CD	CD, MRSA, S. pyogenes	C. diphtheriae	positive	-	tell	-	I	-	S	S	-	-
27	24		Afghanistan	Balkan	yes	lower thigh, knee, upper thigh	CD	CD, MRSA, MSSA, S. pyogenes	C. diphtheriae	positive	positive	tell	-	I	-	S	S	-	-

*UMR unaccompanied minor refugee, MRSA methicillin-resistant *S. aureus*, MSSA methicillin-sensible *S. aureus*, CD *Corynebacterium diphtheria*, coblfos *Columbia blood agar with fosfomycin plates,* tell *tellurite agar,* MIC minimal inhibitory concentration, EUCAST European Committee on Antimicrobial Susceptibility Testing, MLST multilocus sequence typing. Country codes: AL Albania, AT Austria, BA Bosnia and Herzegovina, BG Bulgaria, CH Switzerland, CZ Czech republic, GR Greece, HR Croatia, HU Hungary, IT Italy, IR Iran, IQ Iraq, , MK Macedonia, NL Netherlands, RS Serbia, SI Slovenia, SK Slovakia, TR Turkey, XK Kosovo.

Appendix Table 2. Cases of toxigenic cutaneous infections caused by *Cornyebacterium diphtheria* published over the past 65 years

	Detected isolates of …. in skin infections						
Reference number	toxigenic C. diphtheria (CD)	any CD	C. ulcerans	C. paratuberculosis	MSSA	MRSA	S. pyogenes
(*1*)	1	12	0	0	n/a	n/a	n/a
(*2*)	1	1	0	0	1	0	0
(*3*)	3	3	0	0	1	0	1
(*4*)	2	2	1	0	2	0	2
(*5*)	1	1	0	0	0	0	0
(*6*)	6	13	0	0	n/a	n/a	n/a
(*7*)	14	18	5	0	n/a	n/a	n/a
(*8*)	1	1	0	0	0	1	1
(*9*)	4	4	0	0	1	0	2
(*10*)	2	2	0	0	0	0	2
(*11*)	2	1	0	0	1	0	0
(*12*)	3	3	0	0	2	0	1
(*13*)	0	1	0	0	1	0	1
(*14*)	2	1	0	0	n/a	n/a	n/a
(*15*)	1	1	0	0	1	0	1
(*16*)	0	1	0	0	n/a	n/a	n/a
(*17*)	0	1	0	0	0	1	0
(*18*)	0	1	0	0	1	0	1
(*19*)	10	20	0	0	9	4	15
(*20*)	1	1	0	0	n/a	n/a	n/a
(*21*)	1	1	0	0	0	0	0
(*22*)	0	1	0	0	0	0	0
(*23*)	1	1	0	0	0	0	1
(*24*)	0	0	1	0	0	0	0
(*25*)	0	0	1	0	0	0	0
(*26*)	0	1	0	0	0	0	0
(*27*)	0	1	0	0	1	0	0
(*28*)	0	1	0	0	0	0	0
(*29*)	1	72	0	0	32	3	61
(*30*)	0	0	1	0	0	0	1
(*31*)	1	1	0	0	0	0	1
(*32*)	0	0	1	0	0	0	0
(*33*)	0	1	0	0	0	0	0
(*34*)	0	32	0	0	17	7	22
(*35*)	1	1	0	0	1	0	1
(*36*)	0	33	0	0	18	3	21
(*37*)	0	0	1	0	n/a	n/a	n/a
(*38*)	0	1	0	0	0	0	0
(*39*)	1	5	0	0	n/a	n/a	n/a
(*40*)	6	6	0	0	4	0	4
(*41*)	0	1	0	0	1	0	n/a
(*42*)	3	3	0	0	2	0	1
(*43*)	0	1	0	0	n/a	n/a	n/a
(*44*)	0	0	1	0	1	0	0
(*45*)	0	10	0	0	7	0	6
(*46*)	0	1	0	0	0	0	0
(*47*)	1	1	0	0	1	0	1
(*48*)	1	1	0	0	1	0	0
(*49*)	0	5	0	0	4	0	3
(*50*)	1	1	0	0	0	0	1
**TOTAL**	**72**	**270**	**12**	**0**	**110**	**19**	**151**

References

1. Kofler J, Ramette A, Iseli P, Stauber L, Fichtner J, Droz S, et al. Ongoing toxin-positive diphtheria outbreaks in a federal asylum centre in Switzerland, analysis July to September 2022. Euro Surveill. 2022;27:2200811. https://www.eurosurveillance.org/content/10.2807/1560-7917.ES.2022.27.44.2200811

2. Batista Araújo MR, Bernardes Sousa MÂ, Seabra LF, Caldeira LA, Faria CD, Bokermann S, et al. Cutaneous infection by non-diphtheria-toxin producing and penicillin-resistant Corynebacterium diphtheriae strain in a patient with diabetes mellitus. Access Microbiol. 2021;3:000284 https://www.microbiologyresearch.org/content/journal/acmi/10.1099/acmi.0.000284

3. Marshall NC, Baxi M, MacDonald C, Jacobs A, Sikora CA, Tyrrell GJ. Ten years of diphtheria toxin testing and toxigenic cutaneous diphtheria investigations in Alberta, Canada: a highly vaccinated population. Open Forum Infect Dis. 2022;9:ofab414.

4. Levi LI, Barbut F, Chopin D, Rondeau P, Lalande V, Jolivet S, et al. Cutaneous diphtheria: three case-reports to discuss determinants of re-emergence in resource-rich settings. Emerg Microbes Infect. 2021;10:2300–2.

5. Fuchs F, Markert D, Wagner IV, Liebau MC, Berger A, Dangel A, et al. Toxigenic *Corynebacterium diphtheriae*-associated genital ulceration. Emerg Infect Dis. 2020;26:2180–1.

6. Alberto C, Osdoit S, Villani AP, Bellec L, Belmonte O, Schrenzel J, et al. Cutaneous ulcers revealing diphtheria: a re-emerging disease imported from Indian Ocean countries? Ann Dermatol Venereol. 2021;148:34–9.

7. Gower CM, Scobie A, Fry NK, Litt DJ, Cameron JC, Chand MA, et al. The changing epidemiology of diphtheria in the United Kingdom, 2009 to 2017. Euro Surveill. 2020;25:1900462. https://www.eurosurveillance.org/content/10.2807/1560-7917.ES.2020.25.11.1900462 cited 2023 Feb 14.

8. Govindaswamy A, Trikha V, Gupta A, Mathur P, Mittal S. An unusual case of post-trauma polymicrobial cutaneous diphtheria. Infection. 2019;47:1055–7.

9. Griffith J, Bozio CH, Poel AJ, Fitzpatrick K, DeBolt CA, Cassiday P, et al. Imported toxin-producing cutaneous diphtheria - Minnesota, Washington, and New Mexico, 2015-2018. MMWR Morb Mortal Wkly Rep. 2019;68:281–4.

10. Berger A, Dangel A, Schober T, Schmidbauer B, Konrad R, Marosevic D, et al. Whole genome sequencing suggests transmission of Corynebacterium diphtheriae-caused cutaneous diphtheria in two siblings, Germany, 2018. Euro Surveill. 2019;24:1800683. https://www.eurosurveillance.org/content/10.2807/1560-7917.ES.2019.24.2.1800683 cited 2023 Jan 30.

11. Scheifer C, Rolland-Debord C, Badell E, Reibel F, Aubry A, Perignon A, et al. Re-emergence of *Corynebacterium diphtheriae*. Med Mal Infect. 2019;49:463–6.

12. Fernandez-Pittol M, Bosch J, Muñoz J, Bodro M. Skin infection by Corynebacterium diphtheriae and Streptococcus pyogenes: an unusual association. Enferm Infecc Microbiol Clin. 2019;37:678–9.

13. Lim KHL, Boon M. Cutaneous diphtheria in a returned traveller: cutaneous diphtheria. J Paediatr Child Health. 2019;55:358–61.

14. Edwards D, Kent D, Lester C, Brown CS, Murphy ME, Brown NM, et al. Transmission of toxigenic *Corynebacterium diphtheriae* by a fully immunised resident returning from a visit to West Africa, United Kingdom, 2017. Euro Surveill. 2018;23:1700681. https://www.eurosurveillance.org/content/10.2807/1560-7917.ES.2018.23.39.1700681 cited 2023 Jan 30.

15. Wilson IE, Menson EN. Cutaneous diphtheria. N Engl J Med. 2018;378:e17.

16. du Plessis M, Wolter N, Allam M, de Gouveia L, Moosa F, Ntshoe G, et al. Molecular characterization of *Corynebacterium diphtheriae* outbreak isolates, South Africa, March–June 2015. Emerg Infect Dis. 2017;23:1308–15.

17. Kolios AGA, Cozzio A, Zinkernagel AS, French LE, Kündig TM. Cutaneous *Corynebacterium* infection presenting with disseminated skin nodules and ulceration. Case Rep Dermatol. 2017;9:8–12.

18. Billard-Pomares T, Rouyer C, Walewski V, Badell-Ocando E, Dumas M, Zumelzu C, et al. Diagnosis in France of a non-toxigenic tox gene-bearing strain of *Corynebacterium diphtheriae* in a young male back from Senegal. Open Forum Infect Dis. 2017;4:ofw271.

19. Meinel DM, Kuehl R, Zbinden R, Boskova V, Garzoni C, Fadini D, et al. Outbreak investigation for toxigenic *Corynebacterium diphtheriae* wound infections in refugees from Northeast Africa and Syria in Switzerland and Germany by whole genome sequencing. Clin Microbiol Infect. 2016;22:1003.e1–8.

20. Deshpande A, Inkster T, Hamilton K, Litt D, Fry N, Kennedy ITR, et al. Colonisation with toxigenic *Corynebacterium diphtheriae* in a Scottish burns patient, June 2015. Euro Surveill. 2015;20:30088. https://www.eurosurveillance.org/content/10.2807/1560-7917.ES.2015.20.49.30088

21. Berg L, Mechlin A, Schultz ES. Kutane Diphtherie nach Bagatellverletzung in Sri Lanka. Hautarzt. 2016;67:169–72.

22. Nelson TG, Mitchell CD, Sega-Hall GM, Porter RJ. Cutaneous ulcers in a returning traveller: a rare case of imported diphtheria in the UK. Clin Exp Dermatol. 2016;41:57–9.

23. Sánchez MEG, Alvarez JB, León SH. Chronic nonhealing ulcerated nodules in a Spanish boy after traveling. JAMA Dermatol. 2015;151:1247–8.

24. Moore LSP, Leslie A, Meltzer M, Sandison A, Efstratiou A, Sriskandan S. *Corynebacterium ulcerans* cutaneous diphtheria. Lancet Infect Dis. 2015;15:1100–7.

25. Vandentorren S, Guiso N, Badell E, Boisrenoult P, Micaelo M, Troché G, et al. Toxigenic *Corynebacterium ulcerans* in a fatal human case and her feline contacts, France, March 2014. Euro Surveill. 2014;19:20910. https://www.eurosurveillance.org/content/10.2807/1560-7917.ES2014.19.38.20910

26. Farfour E, Badell E, Jacques Natali L, Dommergues MA, Blot S, Guiso N, et al. Photo quiz: multiple skin lesions on a 9-year-old boy returning from Mali. J Clin Microbiol. 2014;52:3523.

27. FitzGerald RP, Rosser AJ, Perera DN. Non-toxigenic penicillin-resistant cutaneous *C. diphtheriae* infection: a case report and review of the literature. J Infect Public Health. 2015;8:98–100.

28. Jakovljev A, Steinbakk M, Mengshoel AT, Sagvik E, Brügger-Synnes P, Sakshaug T, et al. Imported toxigenic cutaneous diphtheria in a young male returning from Mozambique to Norway, March 2014. Euro Surveill. 2014;19:20835. https://www.eurosurveillance.org/content/10.2807/1560-7917.ES2014.19.24.20835

29. May MLA, McDougall RJ, Robson JM. *Corynebacterium diphtheriae* and the returned tropical traveler. J Travel Med. 2014;21:39–44.

30. Berger A, Boschert V, Konrad R, Schmidt-Wieland T, Hörmansdorfer S, Eddicks M, et al. Two cases of cutaneous diphtheria associated with occupational pig contact in Germany. Zoonoses Public Health. 2013;60:539–42.

31. Berger A, Lensing C, Konrad R, Huber I, Hogardt M, Sing A. Sexually transmitted diphtheria. Sex Transm Infect. 2013;89:100–1.

32. Corti MAM, Bloemberg GV, Borelli S, Kutzner H, Eich G, Hoelzle L, et al. Rare human skin infection with *Corynebacterium ulcerans*: transmission by a domestic cat. Infection. 2012;40:575–8.

33. Orouji A, Kiewert A, Filser T, Goerdt S, Peitsch WK. Cutaneous diphtheria in a German man with travel history. Acta Derm Venereol. 2012;92:179–80.

34. Gordon CL, Fagan P, Hennessy J, Baird R. Characterization of *Corynebacterium diphtheriae* isolates from infected skin lesions in the Northern Territory of Australia. J Clin Microbiol. 2011;49:3960–2.

35. Mina NV, Burdz T, Wiebe D, Rai JS, Rahim T, Shing F, et al. Canada's first case of a multidrug-resistant *Corynebacterium diphtheriae* strain, isolated from a skin abscess. J Clin Microbiol. 2011;49:4003–5.

36. Lowe CF, Bernard KA, Romney MG. Cutaneous diphtheria in the urban poor population of Vancouver, British Columbia, Canada: a 10-year review. J Clin Microbiol. 2011;49:2664–6.

37. Mattos-Guaraldi A, Sampaio J, Santos C, Pimenta F, Pereira G, Pacheco L, et al. First detection of *Corynebacterium ulcerans* producing a diphtheria-like toxin in a case of human with pulmonary infection in the Rio de Janeiro metropolitan area, Brazil. Mem Inst Oswaldo Cruz. 2008;103:396–400.

38. Thappa D, Vetrichevvel T, Pise G, Agrawal K. Cutaneous diphtheria masquerading as a sexually transmitted disease. Indian J Dermatol Venereol Leprol. 2008;74:187.

39. Wren MWD, Shetty N. Infections with *Corynebacterium diphtheriae* : six years' experience at an inner London teaching hospital. Br J Biomed Sci. 2005;62:1–4.

40. Sing A, Heesemann J. Imported Cutaneous Diphtheria, Germany, 1997–2003. Emerg Infect Dis. 2005;11:343–4.

41. Connell TG, Rele M, Daley AJ, Curtis N. Skin ulcers in a returned traveller. Lancet. 2005;365:726.

42. de Benoist AC, White JM, Efstratiou A, Kelly C, Mann G, Nazareth B, et al. Imported cutaneous diphtheria, United Kingdom. Emerg Infect Dis. 2004;10:511–3.

43. von Hunolstein C. Molecular epidemiology and characteristics of *Corynebacterium diphtheriae* and *Corynebacterium ulcerans* strains isolated in Italy during the 1990s. J Med Microbiol. 2003;52:181–8.

44. Wagner J, Ignatius R, Voss S, Höpfner V, Ehlers S, Funke G, et al. Infection of the skin caused by *Corynebacterium ulcerans* and mimicking classical cutaneous diphtheria. Clin Infect Dis. 2001;33:1598–600.

45. Funke G, Altwegg M, Frommelt L, von Graevenitz A. Emergence of Related Nontoxigenic *Corynebacterium diphtheriae* Biotype *mitis* Strains in Western Europe. Emerg Infect Dis. 1999;5:477–80.

46. Hart PE, Lee PY, Macallan DC, Wansbrough-Jones MH. Cutaneous and pharyngeal diphtheria imported from the Indian subcontinent. Postgrad Med J. 1996;72:619–20.

47. Hamour AA, Efstratiou A, Neill R, Dunbar EM. Epidemiology and molecular characterisation of toxigenic *Corynebacterium diphtheriae* var mitis from a case of cutaneous diphtheria in Manchester. J Infect. 1995;31:153–7.

48. Berih A. Cutaneous *Corynebacterium Diphtheriae*: a traveller's disease? Can J Infect Dis. 1995;6:150–2.

49. Gruner E, Opravil M, Altwegg M, von Graevenitz A. Nontoxigenic *Corynebacterium diphtheriae* Isolated from intravenous drug users. Clin Infect Dis. 1994;18:94–6.

50. Antos H, Mollison LC, Richards MJ, Boquest AL, Tosolini FA. Diphtheria: another risk of travel. J Infect. 1992;25:307–10.
